# Structure, Activity and Function of the SETDB1 Protein Methyltransferase

**DOI:** 10.3390/life11080817

**Published:** 2021-08-11

**Authors:** Mariam Markouli, Dimitrios Strepkos, Christina Piperi

**Affiliations:** Department of Biological Chemistry, Medical School, National and Kapodistrian University of Athens, 11527 Athens, Greece; myriam.markouli@gmail.com (M.M.); smd1700150@uoa.gr (D.S.)

**Keywords:** SETDB1, methyltransferase, epigenetics, cancer, schizophrenia, Huntington’s disease, Rett syndrome, Prader–Willi syndrome, congenital heart diseases, inflammatory bowel disease

## Abstract

The SET Domain Bifurcated Histone Lysine Methyltransferase 1 (SETDB1) is a prominent member of the Suppressor of Variegation 3–9 (SUV39)-related protein lysine methyltransferases (PKMTs), comprising three isoforms that differ in length and domain composition. SETDB1 is widely expressed in human tissues, methylating Histone 3 lysine 9 (H3K9) residues, promoting chromatin compaction and exerting negative regulation on gene expression. SETDB1 has a central role in normal physiology and nervous system development, having been implicated in the regulation of cell cycle progression, inactivation of the X chromosome, immune cells function, expression of retroelements and formation of promyelocytic leukemia (PML) nuclear bodies (NB). SETDB1 has been frequently deregulated in carcinogenesis, being implicated in the pathogenesis of gliomas, melanomas, as well as in lung, breast, gastrointestinal and ovarian tumors, where it mainly exerts an oncogenic role. Aberrant activity of SETDB1 has also been implicated in several neuropsychiatric, cardiovascular and gastrointestinal diseases, including schizophrenia, Huntington’s disease, congenital heart defects and inflammatory bowel disease. Herein, we provide an update on the unique structural and biochemical features of SETDB1 that contribute to its regulation, as well as its molecular and cellular impact in normal physiology and disease with potential therapeutic options.

## 1. Introduction

The SET Domain Bifurcated Histone Lysine Methyltransferase 1 (SETDB1) belongs to the family of the Suppressor of Variegation 3–9 (SUV39) proteins [1], representing a member of the group of SET domain-containing protein lysine methyltransferases (PKMTs) which are implicated in epigenetic regulation. SETDB1 is characterized by a highly conserved bifurcated SET domain which contains an intercepting sequence of approximately 150 amino acids [2]. The SET domain was first discovered in the *Suppressor of Variegation 3–9* (*SUV3–9*), *Enhancer of Zeste* (*EZ*) and *Trithorax* genes of *Drosophila* sp. [3]. Subsequent research detected SET domains in more than 40 species, including *Saccharomyces cerevisiae* (*SET1* gene), *S. pombe* (*Clr4^+^* gene) and humans (*SETDB1* as well as other SET domain-containing HKMT genes) [3,4]. Moreover, in *Caenorhabditis elegans,* the *YNCA* gene product exerts high similarity to SETDB1, also containing a bifurcated SET domain. Furthermore, SETDB1 orthologs have been studied in several other species, a few of which are *Mus musculus*, *Rattus norvegicus*, *Danio rerio*, *Bos taurus* and *Macaca mullata,* among others [5].

SETDB1 is involved in a variety of physiologic as well as pathologic processes. Its ability to interact with many different genes at the same time is a product of epigenetic regulation. The term epigenetics is used to describe changes in gene expression without involving alterations in the DNA sequence itself. It includes modifications in proteins called histones, around which the DNA is wrapped, which can influence gene expression by affecting the level of chromatin compaction and, thus, its transcriptional accessibility. Histones form an octamer that is included in the structure of chromatin and is always comprised of histone H2A, H2B, H3 and H4 duplicates. In this way, SETDB1 is able to influence the expression of a multitude of genes by di-/or trimethylating the lysine 9 (K9) residue of the H3 protein located throughout different chromatin regions. It is therefore evident that SETDB1 functions as a chromatin regulator, mediating H3K9 di-/trimethylation. This histone mark functions as a repressive histone post-translational modification by indirectly increasing chromatin’s compaction after recruiting Heterochromatin protein 1 (HP1), thus decreasing its accessibility and affecting gene expression. In more detail, the compacted form of chromatin prevents transcription factors from binding, leading to repression of gene expression or transcription [6]. 

SETDB1 is highly involved in a variety of physiologic functions, interacting with several proteins and transcription factors such as ETS-related gene (ERG) partners to control cell growth and differentiation. In this way, SETDB1 participates in the regulation of important cellular functions. 

A complex interplay of direct and indirect interactions with other enzymes and signaling pathways has been detected to regulate SETDB1 activity in normal cells. However, SETDB1 has also been strongly implicated in the pathogenesis of multiple diseases, including neurological disorders, cardiovascular, gastrointestinal diseases and most notably, tumor development and progression. Tumorigenesis is a great example of this enzyme’s functional variability since SETDB1 may acquire both a tumor-suppressive function in some tissues, as well as an oncogenic function in others, and thus can repress either tumor-suppressing or tumor-promoting genes, respectively.

In the following sections, we provide a detailed description of the structural and biochemical characteristics of SETDB1, addressing its physiologic cell functions and connection with diseases.

## 2. Structural Features of SETDB1

The human *SETDB1* gene (OMIM 604396), alternatively known as ESET, KG1T, KIAA0067, KMT1E or TDRD21, is located on the chromosome 1q21.3 and encodes the SETDB1 protein, composed of 1291 amino acids [7]. The *SETDB1* gene consists of 23 exons and is expressed in several human tissues such as the testes, ovaries, appendix, brain, spleen, lymph nodes and thyroid gland [8].

**Figure 1:**
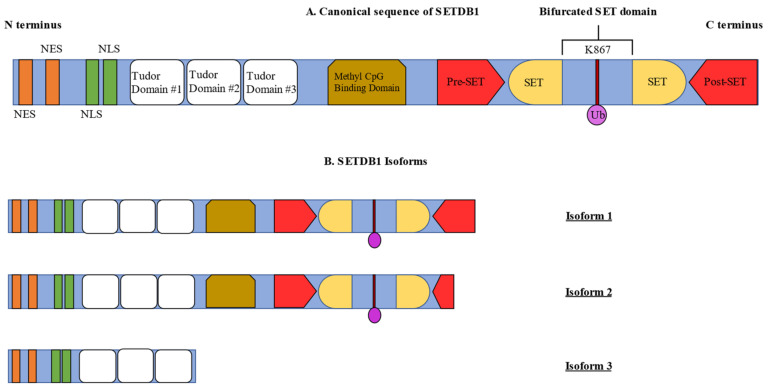
SETDB1 domain composition and isoform architecture. (**A**) The “canonical” sequence of SETDB1 is made up of an N-terminal part which contains two Nuclear Export Signal (NES) domains, two Nuclear Localization Signal (NLS) domains, the three Tudor domains and a Methyl CpG Binding (MBD) domain. The C-terminus of the SETDB1 protein contains the pre-SET, bifurcated SET and post-SET domains. The intercepting sequence of amino acids, which splits the SET domain into two parts, also becomes ubiquitinated at the K867 residue, a post-translational modification that is crucial for the protein’s full functionality. (**B**) SETDB1 exists in three isoforms, only two of which exhibit enzymatic activity. Therefore, isoform 1 is the complete SETDB1 protein, while isoform 2 contains the same domains but is shorter than the first isoform due to alternative splicing. The third isoform lacks all the domains of the C-terminus, exhibiting no enzymatic activity.

The domain composition of SETDB1 includes an N-terminal part which contains three Tudor domains and a methyl-CpG-binding domain (MBD) as well as a C-terminus with a pre-SET, a SET and a post-SET domain [9]. The three Tudor domains are crucial for the formation of complexes with proteins that regulate transcriptional activity via chromatin modifications, such as Histone Deacetylase 1/2 (HDAC1/2) and Kruppel-associated box-Zinc Finger Proteins-KRAB-Associated Protein-1 (KRAB-ZFP-KAP-1) [10] (Figure 1A). This is achieved by the triple Tudor domain binding to H3 tails, which contain the combination of H3K14 acetylation and H3K9 methylation [11]. Moreover, the Tudor domains regulate snRNP processing in Cajal bodies [12]. The MBD domain contains two arginine residues that contribute to DNA binding and is responsible for coupling DNA methylation with H3K9 trimethylation by interacting with DNA Methyltransferase 3 (DNMT3) and inducing gene silencing [13,14]. Additionally, the N-terminal part contains two nuclear export signals (NES) and two nuclear localization signals (NLS), which regulate the localization of SETDB1 [15].

The presence of pre-, post-SET and SET domains at the C- terminus is of paramount importance for the activity of protein methyltransferases. The SET domain is arranged in a helix formation which is linked to an anti-parallel two-stranded β-sheet by loops of different lengths which consist of amino acids that intercept the bifurcated SET domain [16]. This intercepting chain of amino acids, preserved through evolution, was shown to significantly regulate the activity of the SETDB1 protein. The ubiquitination at the lysine residue 867 of SETDB1 mediated by Ubiquitin-Conjugating Enzyme E2 (UBE2E) was demonstrated as a prerequisite for its full methyltransferase activity [17,18]. 

SETDB1 exists in three isoforms, with isoform 1 being considered as the “canonical” sequence, including all the necessary domains for full enzyme activity. Isoform 2 contains the same domains as isoform 1. However, it is produced by alternate splicing of an in-frame splice site which is present in the 3′ coding region, resulting in C-terminal truncation in the post-SET domain, thus producing a shorter protein form. Lastly, isoform 3 lacks all the domains of the C- terminus that are required for enzyme activity (Figure 1B) [19].

## 3. Biochemical Features of SETDB1

The main role of SETDB1 is gene silencing by di- and trimethylating H3K9 residues. In this reaction, SETDB1 utilizes the cofactor S-adenosylmethionine (SAM) as a methyl group donor, which binds to the substrate-binding site of SET [20]. Furthermore, the human homolog of murine ATFa-associated modulator (hAM) is able to induce the conversion of H3 lysine dimethylation to trimethylation and promote the gene-repressive activity of SETDB1 via a SAM-dependent mechanism. This is achieved by binding to SETDB1 thus, forming a SETDB1/hAM complex [21]. The interaction of SETDB1 with hAM, although not a prerequisite for the enzyme’s function, increases its activity [21]. There is evidence that SETDB1 is guided to histone H3 by a factor that is recruited by KRAB zinc-finger proteins, namely, TRIM28/TIF1B. Furthermore, the KRAB–ZFP–KAP-1 complex is also responsible for guiding SETDB1 to H3 in repetitive elements and retrotransposons, as well as other target genes [22,23,24]. This is achieved by KRAB–ZFP interacting with SETDB1 in a sequence-specific manner after SETDB1 interaction with KAP1. KAP1 recruits SETDB1, as well as HP1 and the NuRD histone deacetylase complex [25]. The binding of SETDB1 to H3K9 is achieved via the three Tudor domains, which detect regions of H3 histones containing both K14 acetylation and K9 methylation [11]. Following the methylation of H3K9 by SETDB1, HP1 is recruited to chromatin and alters its structure from a euchromatic to a heterochromatic state [26]. This process is critical for the formation of Heterochromatin since HP1, in turn, works to recruit other proteins, which further establish Heterochromatin formation [27].

Concerning the regulation of SETDB1 expression and activity, several mechanisms have been proposed, which are based on the presence of NES and NLS motifs on SETDB1 protein. These two motifs can control the nuclear levels of SETDB1. Another interaction that can regulate the nuclear levels of SETDB1 is its degradation by the proteasome; however, the importance of this mechanism remains to be explored [28]. Moreover, SETDB1 gains its full enzyme activity after it has been monoubiquitinated at the K867 site, revealing an additional regulatory mechanism [17].

Furthermore, SETDB1 appears to interact with several complexes that can regulate its activity or mediate its functions. In more detail, its activity has been shown to be tightly bound to that of MBD1. SETDB1 can form a complex with MBD1 and ATF7IP, which represses the transcription and couples DNA methylation with H3K9 trimethylation [26,29]. In fact, loss of ATF7IP had similar effects to SETDB1 loss in regards to H3K9 trimethylation and gene transcription levels [30]. MBD1 loss also results in the loss of H3K9me3 in many genomic loci [31]. This indicates that both MBD1 and ATF7IP are crucial for the activity of SETDB1. Although the complete regulatory pathway by which ATF7IP regulates SETDB1 is not fully understood, it has been suggested that ATF7IP contributes to the conversion of H3K9 dimethylation to H3K9 trimethylation by SETDB1 [21]. Further studies have demonstrated that ATF7IP increases the stability of nuclear SETDB1 [30]. The nuclear localization of SETDB1 is regulated by ATF7IP, which binds to the NES motifs and antagonizes their action. Nuclear retention of SETDB1 then upregulates the level of its monoubiquitination, enhancing its activity. Thus, ATF7IP directly acts to ensure SETDB1′s nuclear retention, also promoting its activation while it remains in the nucleus [32]. These results agree with previous studies demonstrating that SETDB1 is mainly located in the cytoplasm of normal and cancer cells while also being able to shuttle between the nucleus and the cytoplasm. Moreover, SETDB1′s transport from the nucleus to the cytoplasm was attributed to the action of Chromosome Region Maintenance 1 (CRM1) nuclear export protein [28], suggesting that the main regulatory mechanisms of SETDB1 activity involve post-transcriptional modifications. In agreement, miRNA targeting of the SETDB1 mRNA has been suggested as an alternative way of post-transcriptional modulation of SETDB1 expression. The miRNA-621, -29 and -381-3p have been shown to downregulate the activity of SETDB1. Interestingly, SETDB1 regulation can also occur on the transcriptional level, and the proteins c-Myc, Specificity Protein 1 (SP1), Specificity Protein 3 (SP3) and TCF4 have been shown to bind to the SETDB1 promoter, inducing its expression [20].

Apart from the abovementioned SETDB1/KAP1/KRAB–Zfp, SETDB1/hAM and SETDB1/DNMT3A/B complexes, SETDB1 can also form large complexes with HDAC1/2, and the transcriptional corepressor mSin3A/B, in order to achieve transcriptional gene repression [33]. In addition, MBD1 attracts SETDB1 to Chromatin assembly factor (CAF-1), thus forming an MBD1/SETDB1/CAF-1 complex, which is specific to the S-phase and facilitates H3K9 methylation and stable Heterochromatin formation [31]. Finally, SETDB1 interacts with the human silencing hub (HUSH) complex, which is required to mediate Heterochromatin formation and gene silencing [21,30]. 

## 4. Physiologic Functions of SETDB1 and Cellular Features

### 4.1. SETDB1 Directly and Indirectly Affects Major Cellular Functions

SETDB1′s cellular functions are mostly related to the trimethylation of H3K9, a repressive mark. Thus, SETDB1 generates a “closed”, more compact and inaccessible chromatin to transcription factors from an “open” and “relaxed”, easily accessible chromatin [10]. SETDB1 can also cause the deposition of other repressive marks on histone tails in an indirect way by cross-talking with other repressive enzymes. One such indirect pathway of gene silencing is the interaction of SETDB1 with the Polycomb Repressive Complex 2 (PRC2), which possesses histone methyltransferase activity and trimethylates Histone 3 Lysine 27 (H3K27), establishing another repressive mark [34]. Therefore, by increasing the enzymatic activity of PRC2, SETDB1 has the potential to interact indirectly with more pathways and repress a wider variety of genes. The change in chromatin architecture caused by SETDB1, especially in gene promoters, leads to the silencing of a vast array of genes. In this way, SETDB1 participates in several cellular functions, including regulation of the cell cycle and cell proliferation [35,36,37,38,39,40], suppression of retroelements [41], regulation of immune cell function [42], maintenance of X chromosome inactivation [28], control of nervous system development [43] and formation of PML-NBs [44]. Additionally, SETDB1 has been implicated in the restriction of pre-adipocyte differentiation [45]. 

### 4.2. SETDB1′s Role in Cell Division

Moreover, it plays a major role in the regulation of cell division and proliferation. Many studies have shown that SETDB1 can interfere with the stability of p53, a central regulator of cell cycle progression and apoptosis, by inhibiting its effects through methylation, thus promoting cell proliferation [46]. Furthermore, SETDB1 has also been linked to increased Protein Kinase B (Akt) activity which, as part of the Inositol trisphosphate (IP3)/Akt pathway, is a crucial regulator of cell proliferation and survival [35]. It has been demonstrated that SETDB1 activates Akt by trimethylating it on the K64 position, leading to TRAF6-mediated Akt ubiquitination in xenograft models [36]. A study by Guo et al. showed that SETDB1 methylates Akt at the K140 and K142 positions, leading to Akt activation, acting in synergy with the PI3K pathway. In this context, the absence of Akt methylation was shown to attenuate its activity [36]. Lastly, the effects of SETDB1 on the cell cycle are attributed to its interaction with central cell cycle regulators, such as Cyclin D1 and c-myc [37].

### 4.3. SETDB1 in ERV Regulation and Its Implications

Another major cellular effect of SETDB1 is the regulation of retroelement expression. Endogenous Retroviruses (ERVs) represent a subcategory of viral retroelements, containing long-terminal repeats which are dispersed among the euchromatic regions of the mammalian DNA [38]. Their transcription has been associated with retrotransposition, a mechanism that causes increased genome instability, leading to many spontaneous mutations [39]. SETDB1 can inhibit ERVs expression, thus minimizing their potential to alter DNA. In accordance, Tan et al. demonstrated that reconstitution of ERV expression in SETDB1-knockout mice upregulates the expression of other neighboring genes. A high percentage of these genes create chimeric transcripts with ERVs or possess ERVs proximally to their initiation sites within a 10 kb range [40]. This role of SETDB1 was observed not only in early embryonic cells but also in further differentiated somatic cells [47]. These results demonstrate that SETDB1 has a continuous role in the repression of ERV expression even after the early developmental stages. Another interesting finding by Fukuda et al. was the connection of SETDB1 with Retroelement Silencing Factor 1 (RESF1), a provirus silencing factor. Their study showed that Resf1 knockout mouse embryonic stem cells had decreased SETDB1 enrichment on provirus and ERV sites. This interaction implicates that RESF1 may also play a part in the SETDB1-mediated repression of ERVs by regulating the action of SETDB1 [48].

A more recent study was able to link the silencing of ERVs by SETDB1 to the regulation of CD4+ T cell differentiation [43]. In more detail, SETDB1 was demonstrated to be essential for both the acquisition and the maintenance of T helper 2 (Th2) response by CD4+ T cells. This study showed that Th2-differentiated, SETDB1-knockdown CD4+ T cells were unable to maintain their Th2 differentiation when exposed to Th1-inducing signals. Takikita et al. demonstrated that SETDB1 is crucial for the selection of single-positive T cells. They observed that SETDB1 deletion resulted in decreased Extracellular Signal-Regulated Kinase (ERK) activity, a protein that is of paramount importance for the development of T cells and is activated by the T Cell Receptor (TCR). This effect was a result of FcγRIIB derepression, which in turn inhibited ERK activation [41]. Finally, SETDB1 has been implicated in the maintenance of primordial germ cells, with decreased SETDB1 activity being associated with depletion of the primordial germ cell pool in males [49]. 

Oogenesis is also influenced by SETDB1, with its depletion in maternal gametes leading to embryonic growth arrest during pre-implantation [50]. The absence of SETDB1 in oocytes induced DNA damage through the reactivation of ERVs, leading to meiosis defects [51]. A similar result was observed in spermatogenic cells, where the loss of SETDB1 resulted in the reactivation of ERVs and caused early meiotic arrest [52].

Lastly, the interaction of SETDB1 with ATF7IP explained above is in part responsible for the maintenance of X chromosome inactivation [26]. It has been shown that depletion of SETDB1 and MBD1 leads to re-expression of Xi genes due to Xi chromosome decompaction, making heterochromatic regions switch into a euchromatic state [26]. Sun et al. found that the decompaction of Xi chromatin upon the loss of SETDB1 was partly due to reactivation of an Endogenous Retrovirus-Related Mammalian-apparent LTR-Retrotransposons (ERVL-MaLR) element and the gene Interleukin 1 Receptor Accessory Protein-Like 1 (IL1RAPL1) [53]. Concerning reproductive system physiology, SETDB1 also seems to be necessary for female identity maintenance in *Drosophila* sp. germ cells since H3K9me3 allows for the suppression of genes normally expressed in testes. SETDB1 loss thus results in the ectopic expression of testicular genes [54]. 

### 4.4. SETDB1 in the Regulation of the Inflammatory Response

In another study, SETDB1 was found to suppress the expression of Toll-Like Receptor 4 (TLR4)-induced proinflammatory mediators, such as IL-6 and IL-12β in macrophages. This effect was attributed to the regulatory effect that SETDB1 had on Nuclear Factor Kappa Beta (NF-κB). Wild-type SETDB1 mice exhibited decreased NF-κB recruitment on the promoter of IL-6, possibly due to the chromatin remodeling effect of H3K9me3. Upon lipopolysaccharide (LPS) stimulation, the H3K9 demethylase LSD2/KDM1B/AOF1 was recruited to promoters of these proinflammatory mediators, decreasing the levels of H3K9me3 and allowing recruitment of NF-κB. This interaction suggests that SETDB1 may act as a gatekeeper for the organism’s inflammatory response, contributing to the balance between repression and activation of proinflammatory cytokines [55].

### 4.5. SETDB1 Regulates the Formation of PML-NBs

Moreover, SETDB1 has been shown to be involved in the formation of PML-NBs, which play a major role in apoptosis, the maintenance of embryonic stem cell pluripotency, DNA damage response and cellular stress as well as tumor growth inhibition. PML-NBs are also involved in the recruitment of proteins such as Death Domain Associated Protein (Daxx), Small Ubiquitin-Like Modifier 1 (SUMO-1), Speckled 100 KDa (Sp100) and CREB-Binding Protein (CBP) [15,56]. A SUMO-interaction motif on the sequence of SETDB1, which binds sumoylated KAP1 and SP3, increases its methylating activity, finally promoting the stabilization of PML-NBs [57]. 

### 4.6. SETDB1 Coordinates the Development of the Nervous System

**Figure 2:**
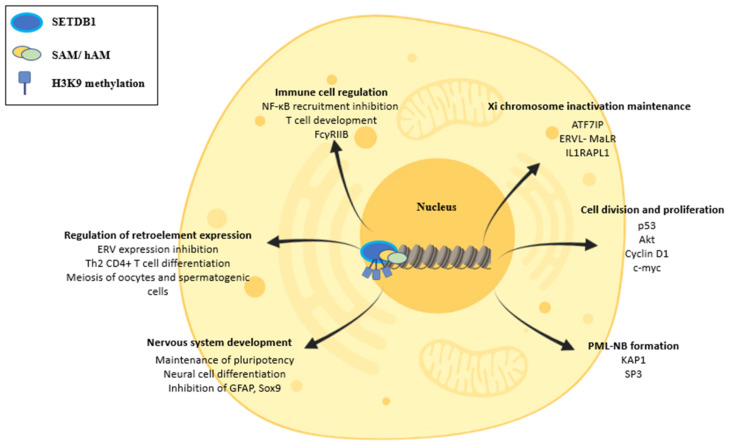
Cellular effects of SETDB1. SETDB1 can trimethylate Histone 3, Lysine 9 (H3K9), using S-Adenosyl methionine (SAM) as a methyl-group donor and human homolog of murine ATFa-associated modulator (hAM) as an inducer, in order to change chromatin composition, inducing compaction of chromatin and gene expression inhibition. In this way, SETDB1 is involved in many cellular functions. It can increase cell division by interfering with p53 and Akt activity, as well as interact with Cyclin D1 and c-myc to induce cell proliferation. SETDB1 can also participate in ERV element suppression, promoting genome stability, differentiation of CD4+ T cells to T helper 2 (Th2) cells and regulation of meiosis in oocytes and spermatogenic cells. In addition, SETDB1 is implicated in immune response regulations by preventing NF-κB recruitment and influencing T-cell development. SETDB1 is also crucial for the formation of PML-NBs. SETDB1 is also critical for the maintenance of X chromosome inactivation (Xi). Lastly, SETDB1 regulates the development of the nervous system by promoting pluripotency and suppressing differentiation markers in early embryogenesis.

On top of the abovementioned physiologic functions, SETDB1 has been demonstrated to participate in the early development of the nervous system. During early embryogenesis, SETDB1 is responsible for maintaining the expression of pluripotency-associated transcription factors while also inhibiting the transcription of trophectoderm differentiation markers [42]. Furthermore, during brain development, SETDB1 takes part in the delicate balance of the amount of neural and astrocytic cells that will be generated. Thus, in the early stages, SETDB1 participates in the inhibition of astrocyte-related genes such as Glial Fibrillary Acidic Protein (GFAP) and SRY box transcription factor 9 (Sox9). In the later stages of brain development, SETDB1 levels decrease, resulting in de-repression of these genes and increased production of astrocytes [40] (Figure 2).

## 5. Connection of SETDB1 with Tumorigenesis

**Figure 3:**
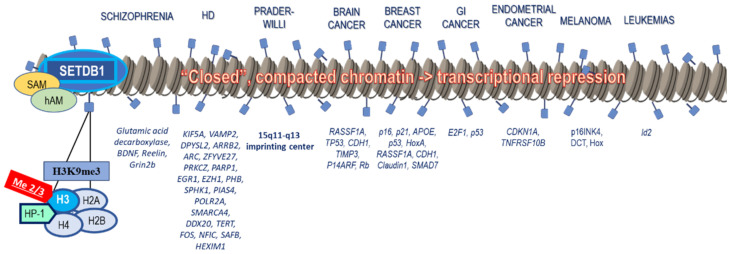
SETDB1 and gene silencing in disease pathogenesis. SETDB1 is capable of epigenetically modifying the Lysine 9 (K9) of histone H3 of the histone octamer through di- or trimethylation. The methyl-group donor during this process is SAM, and hAM works to induce this process. Upon methylation of H3K9 by SETDB1, Heterochromatin Protein (HP1) is recruited, ultimately changing the structure of the chromatin from eu- to Heterochromatin. These changes create a more compact and “closed” chromatin state, which is transcriptionally silenced. Transcriptional inhibition through gene promoter methylation is therefore the main mechanism of SETDB1 implication in a vast array of diseases, and most notably cancer, where SETDB1 is frequently upregulated and mostly serves as an oncogene, silencing tumor suppressor genes.

Aberrant expression and activity of SETDB1 have been implicated in the pathophysiology of various diseases. Most importantly, it has been extensively associated with tumorigenesis. Moreover, it plays an important role in neuropsychiatric and genetic disorders, as well as in some cardiovascular and gastrointestinal diseases (Figure 3).

When it comes to tumorigenesis, SETDB1 downregulates important tumor suppressor genes through histone methylation, acting primarily as an oncogene but also rarely as a tumor suppressor (Figure 3). Another mechanism of SETDB1 involvement in cancer is the suppression of tumor-intrinsic immunogenicity and evasion of the immune response through inhibition of genome regions enriched with transposable elements that would trigger the host’s immune responses if activated [58]. 

### 5.1. Brain and Head–Neck Cancer 

SETDB1 is considered a key mediator of H3K9 trimethylation in CNS tumors and frequently associates with *IDH1* and *BRAF* mutations [39,40]. Increased nuclear SETDB1 expression has been detected in glioma tissues and correlates with high histological grades [44,59,60], as well as enhanced resistance to chemotherapeutic drugs [61]. Elevated SETDB1 expression has also been observed in metastatic head and neck cancers and nasopharyngeal carcinomas, being associated with decreased survival time [62]. SETDB1 upregulation enhances cellular proliferation, invasion and migration, possibly by favoring the transition from the G1 to the S cell cycle phase [63]. It may contribute to brain tumorigenesis by methylating tumor suppressor genes [58], such as *Ras association domain family 1 isoform A (RASSF1A*) [64], *TP53* [65], *E-Cadherin* (*CDH1)* [64], *P14 alternate reading frame (P14ARF)* [64], *Metalloproteinase inhibitor 3* (*TIMP3)* [64] and *Retinoblastoma protein* (*Rb)* [64].

Small interfering RNAs (siRNAs) or histone methyltransferase inhibitors that inhibit SETDB1 have been applied in glioma cell lines and significantly decrease cell proliferation and migration while promoting apoptosis [44,60]. Unfortunately, only non-specific inhibitors against SETDB1 are currently available, including chaetocin, mithramycin A, 3’-deazaneplanocin A (DZNep), paclitaxel (PTX) and miR-381-3p inhibitors [66]. Of note, the chemotherapeutic drug PTX has been shown to downregulate SETDB1 activity through p53 expression and effectively reduce glioma cell growth and brain metastases [67]. Nanoparticle delivery systems are expected to overcome some serious adverse effects, as well as the problem of its low penetration through the Blood–Brain Barrier (BBB), allowing PTX to resurface as a potential therapeutic option against brain cancer.

### 5.2. Lung Cancer and Malignant Pleural Mesothelioma

In Non-Small Cell Lung Cancers (NSCLC), upregulated SETDB1 expression favors tumor progression through interaction and methylation of p53 and Akt (AKT Serine/Threonine Kinase 1) [68,69,70], resulting in poorer prognosis and tumor recurrence in patients with stage I NSCLC [71,72]. According to a recent study, SETDB1-derived circular RNA (circSETDB1) was significantly increased in lung adenocarcinoma hypoxia-induced exosomes and was associated with disease stage, whereas *circSETDB1* knockdown notably inhibited in vitro malignant growth [73]. SETDB1 also activates the Wingless-related integration site (WNT) pathway, causing the accumulation of nuclear β-catenin and induction of a cancerous phenotype [74]. On the contrary, highly metastatic lung adenocarcinomas exhibit decreased SETDB1 activity [75], suggesting that SETDB1 may act as a key oncogene only in the initial stages of NSCLC. SETDB1 targeting in lung cancer has been attempted through the use of the methyltransferase inhibitor DZNep, which downregulates SETDB1 expression and H3K9me3 levels, decreasing lung cancer cell growth and increasing apoptosis [76]. Piperlongumine was also demonstrated to reduce SETDB1 expression, ultimately resulting in the death of lung cancer cell lines [77], along with other chemotherapeutic agents, such as PTX, doxorubicin and cisplatin [66].

The Malignant Pleural Mesotheliomas (MPM) are characterized by a high frequency of SETDB1 mutations, resulting in a non-functional SETDB1 protein [78]. Young-age MPMs often exhibit p53 mutations that lead to chromosomal loss and a near-haploid state, with subsequent genome reduplication and inactivation of SETDB1 [79].

### 5.3. Breast Cancer

Aberrant expression of SETDB1 has been observed in breast cancer (BC) [80], contributing to tumor progression [68]. SETDB1 promotes the Internal Ribosome Entry Segment (IRES)-guided translation of *c-MYC* and *Cyclin D1* (CCND1) oncogenes. Silencing of *SETDB1* drastically decreases the transcription of cell cycle-progression genes, such as phosphorylated *RB, Cyclin A2* and *Cyclin E1*, but also downregulates BMI1, one of the downstream targets of MYC, and increases p21 and p16 expression, contributing to cell cycle arrest and senescence [37]. BC cells lacking SETDB1 exhibited decreased BC type 1 susceptibility protein (BRCA1), a telomere protective and an alternative lengthening of telomeres (ALT)-promoting oncogene involved in the majority of familial BCs [81]. Finally, SETDB1 silencing in BC cells inhibited tumor metastasis through regulation of Mothers against decapentaplegic homolog 7 (SMAD7) expression, which antagonized the transforming growth factor beta (TGF-β) pathway. On the contrary, cells overexpressing SETDB1 were characterized by decreased epithelial markers, such as β-catenin and E-cadherin, but elevated mesenchymal markers, such as vimentin, promoting migration and invasion [82,83]. SETDB1 has been involved in epithelial–mesenchymal transition (EMT) after binding to *Snail* promoter in triple-negative BCs [82]. Hox antisense intergenic RNA (*HOTAIR*), a functional Long Non-Coding RNA (lncRNA), aids in BC progression when overexpressed by indirectly inhibiting miR-7. This causes the upregulation of SETDB1, c-Myc and suppression of E-cadherin, enhancing the EMT process [84,85,86]. Lastly, activated SETDB1 interaction with ΔNp63 in BCs, a p63 isoform without an N-terminal transactivation domain, redirects SETDB1 to specific tumor suppressor genes, such as *p53, Apolipoprotein E (APOE)* and *Homeobox A (HoxA),* causing chromatin changes and gene silencing [87].

Regarding BC treatment, various TGF-β pathway inhibitors target SETDB1, such as SMAD7, which appears to prevent metastasis [83]. Moreover, Cardamonin suppresses SETDB1 and inhibits BC cell growth while also downregulating BC inflammatory mediators that are tied to increased aggressiveness, chemotherapy resistance, poor patient survival and stem cell phenotypes [88]. Lastly, HOTAIR inhibitors, including calycosin, delphinidin-3-glucoside, genistein and BML-284 have been proposed as potential targets in BC treatment [86]. As mentioned above, HOTAIR has been shown to enhance SETDB1, c-myc and STAT3 [84] but suppress E-cadherin [82,89] in favor of EMT [86], thus justifying its potential inhibition in the treatment against BC.

### 5.4. Gastrointestinal Cancers

In CRC, SETDB1 overexpression positively correlates with increased histological grade and stage and associates with poor prognosis [90,91,92]. It contributes to the H3K9 histone methylation of the *p53* promoter, inducing p53 dysregulation in CRC cells [13]. It also binds directly to the Signal Transducer and Activator of Transcription 1 (STAT1) promoter, resulting in the enhanced function of CCND1/Cyclin-dependent kinase 6 (CCND1/CDK6) complex and a shift from the G0/G1 to the S phase [93]. SETDB1 depletion restores the transcriptional status of affected genes back to normal, allowing for cell re-differentiation and cancer cell transformation to normal-like post-miotic cells [94], especially when combined with cytotoxic drugs such as 5-fluorouracil, oxaliplatin and irinotecan.

In Hepatocellular Carcinoma (HCC), SETDB1 upregulation is linked to metastasis and poor prognosis after interaction with p53 and dimethylation of K370 [95,96,97,98]. In pancreatic disease, SETDB1 is needed for exocrine regeneration of the pancreas after cerulein-mediated acute pancreatitis, and its absence leads to significant pancreatic atrophy or apoptosis [99]. In mouse Pancreatic Ductal Adenocarcinoma (PDA), SETDB1 directly binds p53 and regulates its expression. On the other hand, in the early, non-aggressive stages of PDA, SETDB1 may acquire a tumor-suppressive role since it protects cells from KRAS-induced PDA, even after double p53 allele loss. Additionally, SETDB1 deletion induces the accelerated development of pancreatic intraepithelial neoplasia and acinar-to-ductal metaplasia, again suggesting its anti-oncogenic properties. Of importance, overexpression of miR-621, which downregulates SETDB1 and p53 activity, enhanced HCC cell radiosensitivity [100,101], while the H3K9 methylation inhibitor Mithramycin A was shown to significantly reduce HCC tumor growth [36,102,103].

### 5.5. Reproductive System Cancers

Ovarian serous cancer (SOC) is characterized by increased circSETDB1 levels, which are associated with lymph node metastasis, advanced clinical stage, and chemoresistance as well as a shorter patient survival [104,105]. In advanced ovarian cancer, TGF-β- induced epigenetic silencing of epithelial genes, including *CDH1*, is mediated through SETDB1, leading to EMT and metastasis. Additionally, TGF-β activation recruits SMAD2 and 3 to the IL-2 promoter. SETDB1 binding to SMAD3 methylates and suppresses T cell receptor-induced IL-2 transcription, presenting an additional mechanism of SETDB1 involvement in ovarian cancer tumorigenesis [75,106]. 

SETDB1 has also been involved in endometrial carcinoma by inhibiting *p53* and favoring tumorigenesis [107]. SETDB1 is also overly expressed in prostate cancer (PC) tissues, especially when they are androgen-independent. *SETDB*1 knockdown was shown to promote G0/G1 phase arrest, decrease colony formation and suppress cancer growth and migration [108]. It affects genomic stability by interacting with URI (Unconventional Prefoldin RPB5 Interactor protein) that represses retrotransposons [109]. LINE-1 retroelements are derepressed in PC so that URI dysfunction impairs the SETDB1-controlled repressive function of KAP1 on retroelements, favoring genomic rearrangements [110].

### 5.6. Melanoma

In melanomas, SETDB1 is positively correlated with several prognostic factors, including high mitotic counts, advanced invasion depth (Clark levels), involvement of the epidermis and p16INK4 methylation [111,112]. SETDB1 expression was further associated with the *BRAFV600E* mutation in favor of melanoma development [113,114]. The tumorigenic effects of SETDB1 are attributed to the regulation of Thrombospondin 1 (THBS1), which promotes melanoma invasiveness and metastasis as well as downregulation of the expression of DOPAchrome tautomerase (DCT), an enzyme that participates in melanin synthesis [102]. Mithramycin A was shown to reduce SETDB1 expression and tumor growth in melanomas with upregulated SETDB1 levels [36,102,103]. CAS 935693-62-2, a small molecule SETDB1 inhibitor, decreased the number of viable cells overexpressing SETDB1 [111].

### 5.7. Hematologic Cancers

SETDB1 functions as a tumor suppressor in Acute Myeloid Leukemia (AML) by promoter histone methylation and repression of tumorigenic genes [115], such as Sineoculis homeobox homolog 1 (*Six1*), HoxA9 and Dedicator of Cytokinesis 1 (*Dock1)* [116,117]. AML patients exhibit reduced SETDB1 activity, whereas increased SETDB1 levels were positively correlated with more favorable patient survival. However, SETDB1 may be needed for the initiation of AML pathogenesis and early progression since it methylates and represses retrotransposons, thus rescuing AML cells from the patient’s innate immune response that is initiated when sensing retrotransposons as “non-self” [118]. 

Regarding Acute Promyelocytic Leukemia (APL), an aggressive subtype of AML, SETDB1 is a stable member and responsible for the integrity of PML-NBs which are found interspersed in chromatin, regulating transcription, apoptosis and DNA damage responses [119]. SETDB1 regulates PML-NB-associated genes, including Inhibitor of DNA binding 2 *(Id2)*, which is decreased in APL [120,121]. Of note, SETDB1 function may be mediated by the polymerase associated factor (*PAF1*) complex, which regulates *HoxA9* and *Meis1* and other key genes responsible for leukemogenesis [117]. 

The therapeutic potential of SETDB1 inhibition in AML was evidenced by the use of UNC0638, an H3K9me2/3 inhibitor that caused myeloid leukemia cell cytotoxicity, but also cKit+ hematopoietic stem cell line expansion in healthy bone marrow cells [116]. In APL, arsenic trioxide (As_2_O_3_) use in mice resulted in PML degradation, as well as the significant reduction of SETDB1 levels with PML-NBs disassembly and increased Id2 expression [15].

### 5.8. Osteosarcoma

Deletions of the 6q16.3 region of their tumor-suppressor Glutamate Ionotropic Receptor Kainate Type Subunit 2 (*GRIK2*) gene in osteosarcomas have been shown to interfere with SETDB1 binding since this deleted region contains a SETDB1 binding site [122]. SETDB1 normally causes H3K9 methylation and downregulation of *GRIK2* expression. This deletion, therefore, results in overexpression of the anti-oncogenic GRIK2, apoptosis and decreased proliferation and migration while also revealing a possible tumorigenic role of SETDB1 in osteosarcomas [123].

## 6. SETDB1 Connection to Other Diseases

SETDB1 has also been reported to be involved in several other diseases, mostly neuropsychiatric disorders. It is also implicated in a series of genetic diseases, as well as congenital cardiovascular diseases and Inflammatory Bowel Disease (IBD).

### 6.1. SETDB1 Association with Neuropsychiatric Diseases 

SETDB1 plays a major role in the pathogenesis of several neuropsychiatric disorders, such as schizophrenia and autism spectrum disorder, as well as in neurodevelopmental diseases. Increased levels of H3K9me2 and SETDB1 have been found postmortem in patients with a history of schizophrenia, and enhanced methyltransferase activity has been associated with a particular clinical phenotype, consisting of positive family history, longer duration, negative symptoms difficult to treat and thus a poorer disease prognosis [124]. Upregulation of H3K9me2 marks has been observed in schizophrenia biomarker genes, such as *Glutamic acid decarboxylase, Brain-derived neurotrophic factor (BDNF)* and *Reelin* [125,126]. Notably, decreased levels of these biomarkers have also been associated with chronic disease, which bears a worse prognosis and a positive family history. Moreover, SETDB1 was shown to methylate H3K9 in the ventral striatum and hippocampus, thus regulating genes, such as the *NMDA receptor subunit N-methyl D-aspartate receptor subtype 2B (NR2B/Grin2b*). In more detail, *Grin2B* repression is involved in the pathogenesis of schizophrenia and bipolar disorder [127,128]. Lastly, neurodegeneration and memory deficits in Frontotemporal Dementia (FTD) and Amyotrophic Lateral Sclerosis (ALS) are linked to a global downregulation of the H3K9me3 mark, further demonstrating the implication of SETDB1 in the pathogenesis of neuropsychiatric diseases [129]. When it comes to treatment options affecting SETDB1 function in neurocognitive disorders, SETDB1 activity enhancement has anti-depressive effects, and H3K9me3 inhibition with an elevation of BDNF expression was shown to prevent perioperative neurocognitive disorders [130], thus demonstrating the need for further research on the therapeutic modulation of SETDB1 activity.

In neurodevelopment, the impact of SETDB1 is crucial, as evidenced by a de novo 1q21.3 deletion present in patients with intellectual disability (ID) or typical autism spectrum disorder (ASD) that affects the *SETDB1* gene, among others [131]. Additional mutations that impair epigenetic modifications have been detected in patients with ASD, including an in-frame 3 bp deletion in the *SETDB1* gene [132,133]. The influence of SETDB1 on neurodevelopment may further be affected by substances, such as alcohol and nicotine. Alcohol consumption during pregnancy has been shown to lead to dose-dependent fetal epigenetic abnormalities, which result in fetal neurobehavioral deficits in the context of Fetal Alcohol Spectrum Disorder (FASD). SETDB1 is responsible for alcohol-induced epigenetic changes in the fetal DNA. Acute exposure of the fetus to alcohol leads to SETDB1 downregulation, whereas prolonged exposure for more than 7 days increases the enzyme’s levels [134]. In accordance, other studies report significantly upregulated SETDB1 and H3K9me2 levels in the hypothalamus of offspring exposed to alcohol in utero [135]. Administration of choline, which can mitigate the behavioral effects of alcohol exposure [136], can normalize SETDB1 mRNA levels [137], further confirming the association of SETDB1 with the pathogenesis of FASD. On the other hand, human cells exposed to nicotine in vitro demonstrated decreased SETDB1 levels, as well as decreased GLP and G9 methyltransferase levels [138]. The H3K9me2 levels were also downregulated, implying that nicotine may overall be able to antagonize the chromatin-condensing effects of SETDB1.

### 6.2. SETDB1 Association with Genetic Diseases

SETDB1 has also been implicated in the pathophysiology of a series of genetic diseases, such as Huntington’s disease (HD) and Rett, Prader–Willi and Cockayne syndromes. In HD patients, SETDB1 expression is significantly elevated, pointing scientific interest towards approaches that downregulate SETDB1-promoter activity as potential beneficial therapeutic schemes [139]. Experimental studies have shown that Huntingtin (HTT) binds to ATF7IP, a SETDB1 regulator, resulting in low H3K9me3 levels, whereas loss of HTT upregulates H3K9me3 marks mainly on genes affecting neuronal differentiation. Interestingly, genetic variations of *ATF7IP* seem to correlate with HD’s age of onset [140], adding ATF7IP to the list of potential targets for reducing H3K9me3 levels upregulated by the mutant HTT [141]. 

Furthermore, a wide variety of genes largely occupied by H3K9me3 seem to be involved in the pathogenesis of HD, such as synapse-associated genes: *Kinesin heavy chain isoform 5A (KIF5A), Vesicle-associated membrane protein 2 (VAMP2)*, *Dihydropyrimidinase-related protein 2 (DPYSL2)* and *arrestin beta-2 (ARRB2);* cytoskeleton regulation genes: *Activity-regulated cytoskeleton-associated protein (ARC), Zinc finger, FYVE domain containing 27 (ZFYVE27), Protein kinase C, zeta (PRKCZ);* protein metabolism genes: *Poly (ADP-ribose) polymerase 1 (PARP1), Early growth response protein 1 (EGR1), Enhancer of Zeste Homolog 1 (EZH1)* and *Polyhydroxybutyrate (PHB);* immune response genes: *Sphingosine kinase 1 (SPHK1)*, *protein inhibitor of activated STAT protein gamma (PIAS4);* DNA replication and repair genes: *E2F6,RNA polymerase II subunit A (POLR2A), SWI/SNF Related, Matrix Associated, Actin-Dependent Regulator of Chromatin, Subfamily A, Member 4 (SMARCA4), DEAD-box helicase 20 (DDX20)* and *DNA topoisomerase II alpha (TOP2A), Telomerase reverse transcriptase (TERT)* and transcriptional regulation genes: *FOS, Nuclear factor 1 C-type (NFIC) Scaffold attachment factor B (SAFB)* and *Hexamethylene Bis-Acetamide-Inducible Protein 1 (HEXIM1)*. At last, SETDB1 involvement in the HD phenotype was further confirmed with the ocular expression of mHTT in a *Drosophila melanogaster* model with HD, which led to progressive eye degeneration and ommatidium disruption that was exacerbated by SETDB1 overexpression. On the contrary, SETDB1 deletion saved the affected eye [142].

Collectively, increased SETDB1 activity along with the establishment of H3K9me3 marks are suggested to contribute to the suppression of several genes implicated in the pathophysiology of HD. HDAC inhibitors (HDACi) have been shown to improve neuronal survival in HD [143,144] and have been suggested as potential treatment options since the SETDB1 Tudor domain interacts with HDAC 1/2 to achieve transcriptional repression, while mHTT itself reduces the activity of histone acetyltransferases, causing histone deacetylation and thus gene repression [145]. The use of HDACi helps to restore normal transcription and prevents histone deacetylation in the presence of mHTT [145,146]. Examples of HDACi include butyrates nogalamycin, which restores the normal histone H3K9 trimethylation and acetylation balance; cystamine, which decreases *Htt* aggregates and mithramycin, which can induce SETDB1 suppression [147]. The 5-allyloxy-2-(pyrrolidine-1-yl) quinoline (APQ) is another newly discovered SETDB1 inhibitor that reduces H3K9me3 levels and improves motor and neuropathological symptoms in an HD model [148]. Overall, HDAC and SETDB1 inhibitors have both increased HD patient survival, but further research is needed to validate their effects in human clinical trials [142].

SETDB1 is also associated with Rett syndrome, which is caused by mutations in *Methyl-CpG-binding Protein 2 (MECP2)* gene, leading to histone modification dysregulation that causes Heterochromatin formation. *MECP2* knockdown in mice rendered them incapable of tolerating increased H3K9 levels and deteriorated their Rett phenotype, whereas normal mice were able to deal with increased H3K9 methylation, suggesting a possible correlation between Rett syndrome and the H3K9 methylation mark, which is related to SETDB1 activity [149].

Prader–Willi syndrome is a chromosomal deletion of 15q11-q13 of the paternal chromosome, causing a characteristic phenotype that includes intellectual disability, obesity, hyperphagia and hypogonadism [150]. Cruvinel et al. demonstrated that *SETDB1* knockdown in Prader–Willi-specific-induced pluripotent cells (iPSCs) decreased the H3K9me3 levels on the Small Nucleolar RNA 116 (SNORD116) cluster and increased the cluster’s transcriptional activity [151], which is normally silenced in Prader–Willi patients. *SETDB1* knockdown also resulted in decreased methylation of the 15q11-q13 imprinting center, which regulates imprinting [152], but was not able to upregulate the Small Nuclear Ribonucleoprotein Polypeptide N (SNRPN) cluster transcriptional activity, which is implicated in disease pathogenesis. However, the knockdown of the ZNF274 transcription factor, which interacts with SETDB1, decreased the H3K9me3 pattern in the Prader–Willi imprinting center and reactivated both SNORD116 as well as the SNRPN clusters [153]. It is evident that SETDB1 is increased in Prader–Willi and silences the SNORD116 cluster as well as the unaffected maternal chromosome. 

Cockayne syndrome has been associated with *CSA* or *CSB* gene mutations and results in accelerated aging. In cells lacking CSB, unrepaired DNA damage leads to persistent activation of the poly-ADP ribose polymerase (PARP) so that the cell‘s nicotinamide adenine dinucleotide is used and subsequently depleted, resulting in mitochondrial dysfunction. Induction of SETDB1 expression in CSB-deficient cells decreased PAR and restored mitochondrial function. This suggests that CSB defects in Cockayne syndrome are strongly related to SETDB1 downregulation and Heterochromatin loss, allowing for PAR buildup from freely-transcribed regions and thus, mitochondrial dysfunction from freely-transcribed regions and, finally, mitochondrial dysfunction [154].

### 6.3. SETDB1 Association with Cardiovascular Diseases

Studies have shown that disruption of the interaction between SETDB1 and JARID2 could explain how the latter participates in the occurrence of congenital heart defects, such as ventricular septal defect (VSD), double outlet right ventricle (DORV) and hypertrabeculation causing ventricular noncompaction. More specifically, Jarid2 regulates normal cardiac development by silencing *Notch1* through SETDB1 recruitment at its enhancer region, which induces H3K9 trimethylation. Therefore, *Jarid2* deletions in mice resulted in the development of cardiac defects similar to VSD [155], DORV and hypertrabeculation with impaired ventricular compaction, explaining that possible dysregulation of the SETDB1/JARID2 association could form the basis for the development of the abovementioned anomalies [156,157].

### 6.4. SETDB1 Association with Gastrointestinal Diseases 

Rare missense variants of SETDB1 have been identified in Inflammatory Bowel Disease (IBD) patients and are associated with its pathogenesis. Physiologically, SETDB1 participates in intestinal homeostasis, and deletion of *Setdb1* in the intestinal epithelial cells has been shown to impair their differentiation. This results in further loss of transporters responsible for nutrient absorption with subsequent barrier breakdown as well as osmotic fluid shifts, promoting mortality due to metabolic dysfunctions, such as severe dehydration and hypoglycemia. Moreover, *Setdb1* deletion results in de-silencing of ERVs and activation of the innate immune response. Progressive inflammation with DNA damage results in p53 accumulation and intestinal epithelium cell death, further destroying the intestinal barrier but also diminishing the stem cell compartment. Not only deletions but also slight or transient SETDB1 dysregulation due to environmental factors may promote intestinal inflammation. All this justifies the potential implication of the rare *SETDB1* variants observed in IBD patients with disease pathogenesis [158].

## 7. Outlook and Directions for Future Research

Taken altogether, *SETDB1* presents a central regulator of many cellular functions, beginning from early development. Its unique structure with the bifurcated SET domain, as well as the entire structural composition, enable its repressive function, alternating its location between the cytoplasm and nucleus and methylating the H3K9 residues on histone tails to induce chromatin compaction. The collection of cellular effects of SETDB1 make apparent that its role in cell homeostasis is unprecedented, playing a pivotal role in the regulation of the cell cycle along with cell proliferation, the suppression of retroelements which are associated with T cell function, the regulation of immune cell function, the formation of PML-NB bodies, the maintenance of X chromosome inactivation and the development of the nervous system. The array of interacting signaling pathways regulated by SETDB1 has not yet been fully elucidated; however, it has been suggested as a master regulator in many crucial cellular functions.

Aberrant SETDB1 activity has been ultimately linked to disease onset, including nervous, cardiovascular and gastrointestinal system disorders, as well as numerous inherited genetic syndromes. SETDB1 is, however, most significantly involved in tumorigenesis by repressing tumor suppressor genes after establishing the H3K9me3 mark. Altogether, SETDB1 activity results in higher aggressiveness and worse cancer prognosis and has therefore been regarded as an oncogene. Further research on the use of SETDB1 inhibitors to combat aggressive cancer subtypes could help maximize the effects of current therapeutic regimens. First, a deeper understanding of the enzyme’s intracellular effects and affected genes is needed since there is evidence that SETDB1 may also act as a tumor suppressor in some stages of cancer development. The complex interplay of SETDB1 with other epigenetic enzymes also needs more in-depth investigation in order to minimize off-target side effects from its therapeutic targeting. This will allow for the successful implementation of SETDB1 activity manipulations in patient- and disease phenotype-specific treatments to improve patient prognosis and survival rates.

## Data Availability

Not applicable.

## Abbreviations

SET Domain Bifurcated Histone Lysine Methyltransferase 1; SETDB1, Suppressor of Variegation 3-9; SUV39, Protein lysine methyltransferases; PKMTs, Enhancer of Zeste; EZ, H3 lysine 9; H3K9, ETS-related gene; ERG, Methyl-CpG-binding domain; MBD, Histone Deacetylase ½; HDAC1/2, Kruppel-associated box-Zinc Finger Proteins-KRAB-Associated Protein-1; KRAB–ZFP–KAP-1, DNA Methyltransferase 3; DNMT3, Nuclear export signals; NES, Nuclear localization signals; NLS, Ubiquitin-Conjugating Enzyme E2; UBE2E, S-adenosylmethionine; SAM, homolog of murine ATFa-associated modulator; hAM, Heterochromatin protein 1; HP1, Chromosome Region Maintenance 1; CRM1, Specificity Protein 1; SP1, Specificity Protein 3; SP3, Chromatin assembly factor-1; CAF-1, human silencing hub; HUSH, Polycomb Repressive Complex 2; PRC2, Histone 3 Lysine 27; H3K27, Protein Kinase B; Akt, Inositol trisphosphate; IP3, Endogenous Retroviruses; ERVs, Retroelement Silencing Factor 1; RESF1, T helper 2; Th2, Extracellular Signal-Regulated Kinase; ERK, T-Cell Receptor; TCR, Endogenous Retrovirus-Related Mammalian-Apparent LTR-Retrotransposons; ERVL-MaLR, Interleukin 1 Receptor Accessory Protein-Like 1; IL1RAPL1, Toll-Like Receptor 4; TLR4, Nuclear Factor Kappa Beta; NF-κB, lipo-polysaccharide; LPS, Death Domain-Associated Protein; Daxx, Small Ubiquitin-Like Modifier 1; SUMO-1, Speckled 100 KDa; Sp100, CREB Binding Protein; CBP, Glial Fibrillary Acidic Protein; GFAP, SRY Box transcription factor 9; Sox9, Ras association domain family 1 isoform A; RASSF1A, E-Cadherin; CDH1, P14 alternate reading frame; P14ARF, Metalloproteinase inhibitor 3; TIMP3, Retinoblastoma protein; Rb, Small interfering RNAs; siRNAs, 3’-deazaneplanocin A; DZNep, paclitaxel; PTX, Blood–Brain Barrier; BBB, Non-Small Cell Lung Cancers; NSCLC, SETDB1-derived circular RNA; circSETDB1, Wingless-related integration site; WNT, Malignant Pleural Mesotheliomas; MPM, breast cancer; BC, Internal Ribosome Entry Segment; IRES, Cyclin D1; CCND1, BC type 1 susceptibility protein; BRCA1, alternative lengthening of telomeres; ALT, Mothers against decapentaplegic homolog 7; SMAD7, transforming growth factor beta; TGF-β, epithelial mesenchymal transition; EMT, Hox antisense intergenic RNA; HOTAIR, Long Non-Coding RNA; lncRNA, Apolipoprotein E; APOE, Homeobox A; HoxA, Signal Transducer and Activator of Transcription 1; STAT1, CCND1/Cyclin-dependent kinase 6; CCND1/CDK6, Hepatocellular Carcinoma; HCC, Pancreatic Ductal Adenocarcinoma; PDA, Ovarian serous cancer; SOC, Prostate cancer; PC, URI; Unconventional Pre-folding RPB5 Interactor protein, Thrombospondin 1; THBS1, DOPAchrome tautomerase; DCT, Sineo-culis homeobox homolog 1; Six1, Dedicator of Cytokinesis 1; Dock1, Acute Promyelocytic Leukemia; APL, Polymerase associated factor; PAF1, arsenic trioxide; As2O3, Glutamate Ionotropic Receptor Kainate Type Subunit 2; GRIK2, inflammatory bowel disease; IBD, brain-derived neurotrophic factor; BDNF, N-methyl D-aspartate receptor subtype 2B; NR2B/Grin2b, frontotemporal dementia; FTD, Amyotrophic Lateral Sclerosis; ALS, Brain-derived neurotrophic factor; BDNF, intellectual disability; ID, autism spectrum disorder; ASD, fetal alcohol spectrum disorder; FASD, Huntington’s disease; HD, Huntingtin; HTT, Kinesin heavy chain isoform 5A; KIF5A, Vesicle-associated membrane protein 2; VAMP2, Dihydropyrimidinase-related protein 2; DPYSL2, arrestin beta-2; ARRB2, activity-regulated cy-toskeleton-associated protein; ARC, Zinc finger FYVE domain containing 27; ZFYVE27, Protein kinase C zeta; PRKCZ), Poly (ADP-ribose) polymerase 1; PARP1, early growth response protein 1; EGR1, Enhancer Of Zeste Homolog 1; EZH1 and Polyhy-droxybutyrate; PHB, Sphingosine kinase 1; SPHK1, protein inhibitor of activated STAT protein gamma; PIAS4, RNA polymerase II subunit A; POLR2A, Subfamily A, Member 4; SMARCA4, DEAD-box helicase 20; DDX20, DNA topoisomerase II alpha; TOP2A, Telomerase reverse transcriptase; TERT, Nuclear factor 1 C-type; NFIC, Scaffold attachment factor B; SAFB, Hexamethylene Bis-Acetamide-Inducible Protein 1; HEXIM1, HDAC inhibitors; HDACi, Methyl-CpG-binding Protein 2; MECP2, Small Nucleolar RNA 116; SNORD116, Small Nuclear Ribonucleoprotein Polypeptide N; SNRP, poly-ADP ribose polymerase; PARP, ventricular septal defect; VSD, double outlet right ventricle; DORV.
